# A Step in Advance

**Published:** 1921-03

**Authors:** Julio Endelman


### A STEP IN ADVANCE

BY JULIO ENDELMAN, D.D.S.

The Hearst publications of California undertook some time ago and have now well under way a campaign of educating the public in matters pertaining to the care of the teeth and the relation of diseased teeth to systemic health. Editorials and special articles have been appearing almost daily and have been read by thousands in every corner and nook of the state. This campaign is commendable and unique in various respects. It is directed at the public in general, with the end in view that its appreciation of the functions of the teeth may be more definite and their influence as etiologic factors of metastatic infections may be more clearly understood. The editorials are carefully prepared and bring out emphatically just such facts as the dental profession has been for years endeavoring to place altruistically before the public. In addition, this campaign is to bear fruit in moulding public opinion to a better realization of the need of thorough dental services in the public schools, as a state prerogative. The dental services which are being rendered in our public institutions are inadequate and incomplete and the larger percentage of dental services dispensed to children are of an emergency and radical character. If boards of education throughout the state will view the problem of defective teeth from an economical standpoint, to say nothing of its humanitarian aspect, we feel certain that in a short time dental and medical inspection and treatment will assume a degree of importance in the public school curriculum second to none. The parents and guardians of children are not deserving of the blame attached to them for the neglect of the care of the little ones' teeth. They have been kept in total ignorance concerning the preservation of the teeth. At most one-fifth of the population can afford to pay for dental services and, incidentally, receive valuable suggestions as to the care of the teeth. The remaining four-fifths are in complete obscurity concerning mouth hygiene, and when at last, through no longer bearable pain they seek help, the mutilation of the mouth by extractions, or the sowing of the seeds of future invalidism through concealed infections closes, although temporarily only, tragic periods in their existences.

If the public-school system has for its object the development of mental power in order that the children of today may become the valuable citizens of tomorrow, why, we ask, should this educational structure be erected, in perhaps as high as 90 per cent. of the cases, upon a weak body foundation through diseased teeth, tonsils, mucous membranes and all the general infections traceable to these sources?

Voluminous statistical literature on the havoc wrought by dental caries and all its complications is available in dental and medical journals. The story of the thousands upon thousands of children who at maturity have to face the battle of life with only a share of the mental and physical efficiency which should be theirs *in toto,* has been recorded time and again. Ignorance of these facts is not justifiable and neglect to provide the means to lessen the incidence of these diseases—*ergo,* to help boys and girls to reach vigorous manhood and womanhood—can not be viewed in any light other than that of criminal negligence.

The dental profession, which of all professions is the one most vitally interested in spreading the gospel of good teeth and of health conservation through prevention scientifically applied, acclaims the dental campaign of the Hearst publications as one pregnant with untold possibilities for racial improvement, both physical and mental.

Two examples of the excellent editorials which the

Los Angeles' *Examiner* has published are herewith reprinted:

SOUND TEETH IN SCHOOLS IS A VITAL CIVIC NEED

If hookworm were as prevalent among us as bad teeth, there would be a public scare, and a public demand for relief from its ravages from end to end of the state.

Among many useful lessons taught the American people in the war, we have learned, via the government's physical examinations of men for military service, that we are a nation of defectively teethed units, at least to the extent of sixty-odd per cent. or more. The development of this startling phenomenon has led to scientific investigation and a diffusion of public knowledge on the subject, and now nearly everybody knows that it accounts for a fearful sum of physical and mental deficiency, discomfort, pain, discontent and shortened life.

Pyorrhea is not a new thing, but knowledge of its presence, character and agency in spreading poison throughout the human body and causing or inviting no end of lethal diseases, is new. Always it has been with us, only we haven't realized the fact, because it has been hidden, like the 'possum, in the other gum tree.

When we speak of American bad teeth, the term does not seem to connote a national or local question of vital economic, political and moral importance; but it is all that. Our only plant for the production of patriotic, intelligent, efficient and useful citizens is the public school. We can not make the product pass inspection if the warp and woof of the raw material be rotten in any essential spot. Not to extend argument, it can not be doubted that the prevailing evil of bad teeth among our young of school age, if left unremedied, seriously would obstruct the objects and purposes of public education.

Our educational authorities are alive to this danger. Our other public officials and citizens must wake up to it.

The *Examiner* news columns have shown the needs of our schools in the matter of more dentists and dental materials. In both they are sadly lacking.

In the first, last and middle analysis, the good teeth problem is the business of the state, city and every citizen.

SCHOOL DENTAL CLINIC MUST HAVE MORE SUPPORT

Only recently has it dawned upon the civilized world that many of the ills and ailments afflicting advanced nations are due to neglect of teeth in childhood. This neglect results in many specific diseases, such as tonsilitis, infant paralysis and a poisonous condition of the whole body in varying degree and extent, but always dangerous.

Not so long ago even well-educated dentists and physicians entertained a vague and unscientific idea that bad teeth in children were inherited from parents similarly afflicted. Now it is known that they are not hereditary, but due to neglect and bad dietetic habits in the early years of life.

Defective teeth among our public-school pupils mean already poisoned bodies, and common symptoms are toothache, earache, poor sight, indigestion, liver and kidney disturbances, and many other deficiencies formerly never traced to the little ivory guardians of mankind's front door.

It can not be expected that brains will function properly under such physical conditions. Capacity to study, learn or to retain anything learned, sadly is depreciated. In trying to cultivate minds defected by infected bodies the community is in danger of throwing away its money.

Our very intelligent and thorough educational authorities and leaders of thought fully comprehend the nature, dangers and difficulties of the problem. They are quite as alive to the means necessary to a solution as are like officials and thinkers in the large Atlantic cities, where generous relief has been and is being supplied extensively and increasingly by means of adequate and completely manned and equipped dental clinics.

Here, in the metropolis of the great Southwest, we have made a start and that is about all. Between a start and a finish there are several quarter posts, and as yet we have not turned the first one.

During the past school year three overworked dentists attended to 2,446 patients in 12,825 sittings. Today the demand for attention is far ahead of the clinic's ability to give it. A long waiting list is the result, and schools have been warned not to add to it for the next two weeks.

Hundreds of children who are unable to resort to other than free clinics thus are left to suffer while they wait. The law, unfortunately, compels the school authorities to charge their young patients for the materials used.

The Los Angeles Public School Dispensary and Clinic, at 936 Yale Street, is doing the best to be expected from its limited means, accommodations and staff. Now is the time to act.

Heretofore the *Examiner* has commented on the munificent gifts and endowments that wealthy and public-spirited citizens in Eastern cities have devoted to this object in their home places. Perhaps its patriotic, economic and practical appeal may produce an exhibition of the same spirit here.

Nothing is too good for our children of tender age and nothing more important than their health. It ranks with morals.—*Pacific Gazette.*

